# Statistical findings and outcomes of acute coronary syndrome patients during COVID-19 pandemic: A cross sectional study

**DOI:** 10.1016/j.ijcha.2023.101213

**Published:** 2023-04-26

**Authors:** Ferdy Sanjaya, Miftah Pramudyo, Chaerul Achmad

**Affiliations:** Department of Cardiology and Vascular Medicine, Universitas Padjadjaran – Hasan Sadikin General Hospital, Indonesia

**Keywords:** ACS, COVID, In-hospital delay, Mortality

## Abstract

**Introduction:**

Time to treatment of acute coronary syndrome (ACS) can be a matter of life or death considering its major contribution to cardiovascular mortality. The sudden outbreak of the Coronavirus Disease in 2019 (COVID-19) caused great uncertainty in achieving ACS time-frame goals. This study assesses ACS presentation time and outcomes before and during the COVID-19 pandemic.

**Methods:**

A total of 1287 ACS patients were included in this cross-sectional study. We compared mortality and other outcomes during hospital admission. Before-COVID was deemed as admission between March 2018 and February 2020, while admission between March 2020 and February 2022 was deemed as during-COVID. The association of admission on outcomes was measured using regression statistics.

**Results:**

There was a 51.2 % decline of total patients before-COVID (865 patients) to during-COVID (422 patients). While there is no difference in first medical contact (FMC) before [3 h (IQR 1–7)] compared to during the pandemic [3 h (IQR 2–9), p 0.058], we found a decrease in door to wire time < 12 h (43.41 % vs 18.98 %, p < 0.001). There was also a non-significant decrease in fibrinolysis (20.45 % vs 15.18 %, p 0.054) but an increase in those undergoing percutaneous coronary intervention (PCI) (58.36 % vs 77.04 %, p value < 0,001). We also found reduced mortality (12.52 % vs 9.69 %, p 0.151), heart failure (28.16 % vs 25.81 %, p 0.31), but more cardiogenic shock during the pandemic (9.19 % vs 13.33 %, p 0.028).

**Conclusions:**

While the mortality seems statistically unaffected, we found less admission and prolonged door to wire time during-COVID pandemic.

## 1 Introduction

Cardiovascular disease, especially ACS had a great burden and impact in society [1]. After the findings of thrombus as a culprit in coronary artery by Dewood et al, strategies of reperfusion changes from contemplation to intervention [2]. Even after the proven superiority of PCI compared to fibrinolysis, the selection and implementation of reperfusion strategy in real world remained worthy of discussion [3], [4]. Numerous clinical trials have demonstrated that early presentation and shorten door to balloon time was associated with decreased mortality [5]. A similar concept can be attributed to high and very high risk patients with Non ST elevation Acute Coronary Syndrome (NSTEACS), highlighting the importance of invasive strategy [6]. Unfortunately, PCI is not always immediately available, and fibrinolysis, if not contraindicated, should be initiated in ST elevation myocardial infarction (STEMI) [3].

Along with intra-hospital delays, patient-related factors can contribute to delayed treatment either due to psychological, geographical, or logistical factors, some of which became apparent and significantly worsened as a result of the sudden outbreak of COVID-19 [7]. Up to August 2022, there were >500 million cumulative COVID-19 cases globally. Indonesia, the location of this study, ranked 18th globally for COVID-19 infections, showing great burden to health care management [8].

COVID-19 can indirectly affect ACS treatment contributing to patient’s delay resulted in prolonged FMC or directly increase mortality as the direct effect of the virus. Spaccarotella et al highlighted the delay and decreased hospitalization in Italy and northern California compared to the non-COVID-19 periode [9]. Several potential reasons including reluctance of patients to visit hospital to avoid COVID-19 exposure has been considered. In addition, “stay at home” campaigning from the government and media to contain the infection would have delayed hospital presentation for ACS [10]. However, the impact of such decision still under highlight.

European Society of Cardiology (ESC) guidance for the diagnosis and management of cardiovascular disease during the COVID-19 pandemic still prioritized primary PCI with an addition of 60 min delayed time due to COVID-19 related screening procedure [11]. However, this target is difficult to achieve as there were different protocols in local guideline for COVID-19. More recently, COVID-19 was considered endemic in many countries, however thousands of cases were still diagnosed in many countries, including Indonesia [12]. We assessed presentation times and outcomes in real world data to compare the ACS outcomes before and during the COVID-19 pandemic.

## 2 Material and methods

### 2.1 Study population

This cross-sectional study used medical record data from all patients with ACS who were admitted to Hasan Sadikin General Hospital (RSHS) from March 2018 to February 2022. The study was reviewed and approved by the RSHS ethics board. Based on their admission period, 865 patients were admitted before the COVID pandemic and 422 afterwards. Patients who did not have a precise ACS diagnosis, incomplete medical record or positive COVID-19 swab test are excluded from this study (64 patients). The study collects data on age, gender, mortality status, cardiogenic shock status, congestive heart failure status, significant arrhythmias such as total AV block (TAVB) or ventricular tachycardia (VT)/ ventricular fibrillation (VF), ACS diagnosis, fibrinolysis treatment, PCI status, FMC time, duration between ACS onset until intervention, history of previous hypertension, diabetes mellitus (DM), body mass index (BMI) based on Asia Pacific classification, and Killip class on admission. ACS status is divided into two groups which are STEMI and NSTEACS. The BMI classification was divided into four groups which are underweight (<18.5 kg/m^2^), normal (18.5–23 kg/m^2^), overweight (23–24.99 kg/m^2^) and obese (≥25 kg/m^2^).

### 2.2 Statistical analysis

These variables were then compared based on the patient’s admittance period, which is divided into before and during COVID-19 pandemic to assess whether there were significant differences to these characteristics due to changes in standard medical procedure regarding COVID-19 testing. Missing data were excluded from statistical analysis. Categorical variables were compared using chi-square analysis. Normally distributed numeric data was analysed with independent T-Test and Mann-Whitney was used for numeric data that was not normally distributed. Significant difference was stated if the p-value was<0.05. We then also performed multivariate analysis using logistic regression method to ascertain the influence of pandemic periods on the outcomes of interest, which were mortality, cardiogenic shock, congestive heart failure and arrythmias. The model that was used includes variables such as age, gender, previous history of diabetes, hypertension, smoking, dyslipidemia and familial history of myocardial infarction. Using this model, we analysed the period effects after controlling it by including the aforementioned variables that were known to affect the study's outcomes.

## 3 Results

**Supplementary figure 1:**
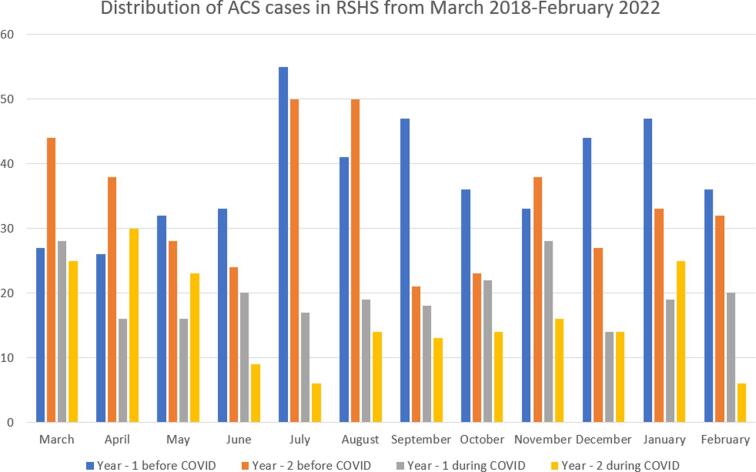
Distribution of ACS cases in Hasan Sadikin General Hospital from march 2018 to February 2022.

**Supplementary figure 2:**
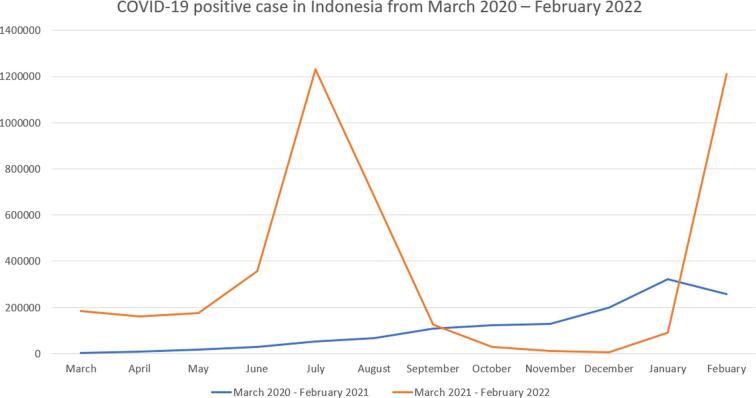
Distribution of COVID-19 positive cases in Indonesia from March 2020- February 2022^12^.

This study includes 1287 patients diagnosed with ACS from March 2018 to February 2022. March 2018 to February 2020 was defined as before-COVID and March 2020 to February 2022 was defined as a during-COVID. There was a 51.2  % (443 patients) decline of total patients from before-COVID (865 patients) to during-COVID (422 patients). The monthly distribution showed that there were less admissions when there was an increase in national COVID-19 cases. (Supplementary Figure 1 & 2).

**Fig. 1:**
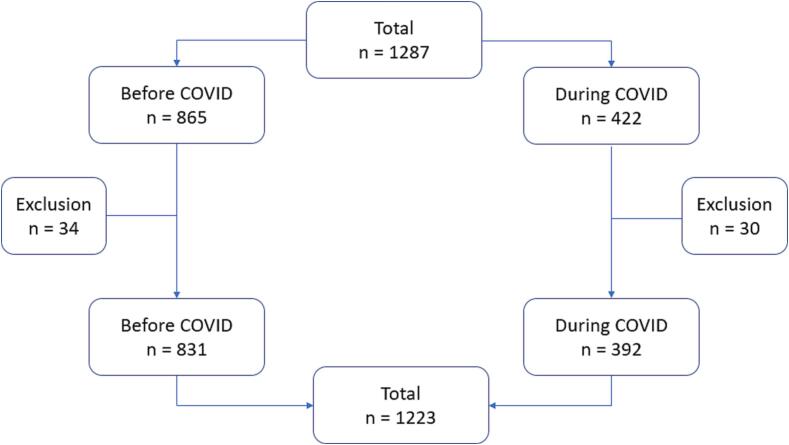
Total population included in this study.

We excluded 34 patients from before-COVID and 30 patients from during-COVID (Fig. 1). The majority of patients were male, the mean age was 58.25 years before-COVID (SD 10.56) and 57.95 years during-COVID (SD 11.88). There was statistically increased (p value 0,001) in percentage of Killip class I (70.4 % vs 73.72 %) and class IV (9.15 % vs 13.27 %) while a decrease was observed in class II (16.61 % vs 10.97 %) and class III (3.37 % vs 1.53 %). There was a significant decrease in proportion of patients with dyslipidemia, smoker, and overweight during-COVID-19 while other risk factors showed similarity statistically. Although we found a decline in patient undergoing fibrinolytic during pandemic (20.45 % vs 15.18 %, p value 0.054), we observed a significant increase in PCI (58,36 % vs 77.04 %, p value < 0,001). Similar results were seen in STEMI and NSTEACS populations. Interestingly, although we expected an increase, we actually found reduced mortality during-COVID (12.52 % vs 9.69 %, p 0.151). We also found reduced heart failure (28.16 % vs 25.38 %, p 0.31) but observed more patient with cardiogenic shock during-COVID (9.19 % vs 13.33 %, p 0.028) (Table 1).

**Table 1:** Baseline Characteristics.

Characteristics	Before-COVID (N = 831)	During-COVID (N = 392)	P value
Age, mean (SD)	58.25 (10.56)	57.95 (11.88)	0.665
STEMI, N (%)	58.16 (10.66)	56.35 (12.06)	0.044
NSTEACS, N (%)	58.33 (10.46)	60.18 (11.37)	0.071
Male gender, N (%)	639 (76.99)	304 (77.55)	0.827
STEMI, N (%)	386 (79.1)	187 (83.48)	0.171
NSTEACS, N (%)	251 (74.04)	117 (70.48)	0.398
Total cases			
STEMI, N (%)	489 (58.84)	224 (57.14)	0.592
NSTEACS, N (%)	339 (40.79)	166 (42.35)	
PCI, N (%)	485 (58.36)	302 (77.04)	<0.001
STEMI, N (%)	321 (65.64)	184 (82.14)	<0.001
NSTEACS, N (%)	163 (48.08)	118 (71.08)	<0.001
Fibrinolytic, N (%)			
STEMI, N (%)	100 (20.45)	34 (15.18 %)	0.054
Dyslipidemia, N (%)	169 (20.34)	53 (13.52)	0.004
STEMI, N (%)	85 (17.38)	16 (7.14)	<0.001
NSTEACS, N (%)	84 (24.78)	37 (22.29)	0.527
Diabetes, N (%)	178 (21.42)	79 (20.15)	0.628
STEMI, N (%)	87 (17.79)	39 (17.41)	0.892
NSTEACS, N (%)	88 (25.96)	40 (24.1)	0.665
Hypertension, N (%)	531 (63.90)	233 (59.44)	0.162
STEMI, N (%)	293 (59.92)	118 (52.68)	0.069
NSTEACS, N (%)	236 (69.62)	114 (68.67)	0.904
Current smoker, N (%)	524 (63.06)	184 (46.94)	<0.001
STEMI, N (%)	336 (68.71)	132 (58.93)	0.009
NSTEACS, N (%)	187 (55.16)	52 (31.33)	<0.001
Overweight, N (%)	288 (34.66)	79 (20.15)	<0.001
STEMI, N (%)	175 (35.79)	53 (23.66)	0.064
NSTEACS, N (%)	112 (33.04)	26 (15.66)	0.001
Obesity, N (%)	279 (33.57)	154 (39.29)	<0.001
STEMI, N (%)	164 (33.54)	81 (36.16)	0.064
NSTEACS, N (%)	114 (33.63)	72 (43.37)	0.001
Family history of premature CAD, N (%)	82 (9.87)	42 (10.71)	0.642
STEMI, N (%)	46 (9.41)	21 (9.38)	0.989
NSTEACS, N (%)	34 (10.03)	21 (12.65)	0.381
Killip class, N (%)			
I	585 (70.4)	288 (73.48)	0.001
II	138 (16.61)	43 (10.97)	
III	28 (3.37)	6 (1.53)	
IV	76 (9.15)	52 (13.27)	
Death, N (%)	104 (12.52)	38 (9.69)	0.151
STEMI, N (%)	67 (13.7)	21 (9.38)	0.103
NSTEACS, N (%)	36 (10.62)	16 (9.64)	0.733
Cardiogenic shock	76 (9.19)	52 (13.33)	0.028
STEMI, N (%)	61 (12.5)	38 (16.96)	0.110
NSTEACS, N (%)	15 (4.46)	14 (8.48)	0.07
Heart failure	234 (28.16)	99 (25.38)	0.310
STEMI, N (%)	135 (27.61)	57 (25.45)	0.546
NSTEACS, N (%)	97 (28.61)	42 (25.45)	0.456

CAD: Coronary Artery Disease; COVID: Corona Virus Disease; NSTEACS: Non-ST Elevation Acute Coronary Syndrome; PCI: Percutaneous Coronary Intervention; SD: Standard Deviation; STEMI: ST Elevation Myocardial Infarction.

We found a similar FMC before-COVID compared to during-COVID (3 h (IQR 1–7) vs 3 h (IQR 2–9), p value 0.058) either in sub group of STEMI or NSTEACS. We also found a significant increase in FMC patient who got fibrinolytics after 6 h (9 % vs 16.67 %, p 0.012). (Table 2).

**Table 2:** First medical contact before and during COVID.

Characteristics	Before-COVID (N = 831)	During-COVID (N = 392)	P value
FMC (Hours), Median (IQR)	3 (1–7)	3 (2–9)	0.058
STEMI, N (%)	2.5 (1–6)	3 (1–8)	0.543
NSTEACS, N (%)	3.5 (1–10)	4 (2–12)	0.591
FMC (all cases)			
0–2 h, N (%)	376 (45.25)	148 (36.72)	Ref
2–6 h, N (%)	217 (26.11)	93 (23.08)	0.589
6–12 h, N (%)	102 (12.27)	52 (12.90)	0.186
12–48 h, N (%)	84 (10.11)	39 (9.68)	0.446
>48 h, N (%)	35 (4.21)	22 (5.46)	0.103
FMC (STEMI)			
0–2 h, N (%)	238 (48.67)	97 (43.30)	Ref
2–6 h, N (%)	135 (27.61)	51 (22.77)	0.710
6–12 h, N (%)	58 (11.86)	30 (13.39)	0.350
12–48 h, N (%)	34 (6.95)	17 (7.59)	0.523
>48 h, N (%)	16 (3.27)	11 (4.91)	0.198
FMC (NSTEACS)			
0–2 h, N (%)	138 (40.71)	50 (30.12)	Ref
2–6 h, N (%)	81 (23.89)	42 (25.30)	0.154
6–12 h, N (%)	44 (12.98)	22 (13.25)	0.296
12–48 h, N (%)	49 (14.45)	21 (12.65)	0.586
>48 h, N (%)	19 (5.6)	11 (6.63)	0.254
FMC (PCI)			
0–2 h, N (%)	228 (47.01)	121 (38.91)	Ref
2–6 h, N (%)	123 (25.36)	74 (23.79)	0.498
6–12 h, N (%)	60 (12.37)	39 (12.54)	0.387
12–48 h, N (%)	47 (9.69)	25 (8.04)	0.993
>48 h, N (%)	18 (3.71)	12 (3.86)	0.557
FMC (Fibrinolytics)			
0–2 h, N (%)	54 (54)	13 (36.11)	0.012
2–6 h, N (%)	33 (33)	5 (13.89)	
6–12 h, N (%)	9 (9)	6 (16.67)	

COVID: Corona Virus Disease; FMC: First Medical Contact; NSTEACS: Non-ST Elevation Acute Coronary Syndrome; PCI: Percutaneous Coronary Intervention; STEMI: ST Elevation Acute Myocardial Infarction.

There was an increase ratio for PCI during pandemic (OR 2.844, p < 0.001); however, we also noticed increased odd ratio in door to wire time after 12 h during pandemic especially in the STEMI subgroup (Table 3).

**Table 3:** Comparison of PCI and Door to Wire Time between Periods.

		Before-COVID		During-COVID		OR	P value
PCI	All cases	485	58.36 %	302	77.04 %	2.844	<0.001
	STEMI	321	65.64 %	184	82.14 %	2.611	<0.001
	NSTEACS	163	48.08 %	118	71.08 %	3.365	<0.001
Door to Wire (All cases)	< 12 h	214	43.41 %	56	18.98 %	Ref	<0.001
	12–48 h	129	26.17 %	110	37.29 %	3.259	
	> 48 h	150	30.43 %	129	43.73 %	3.286	
Door to Wire (STEMI)	< 12 h	178	54.43 %	43	23.37 %	Ref	<0.001
	12–48 h	79	24.16 %	71	38.59 %	3.72	
	> 48 h	70	21.41 %	70	38.04 %	4.14	
Door to Wire (NSTEACS)	< 12 h	36	21.82 %	13	11.71 %	Ref	0.094
	12–48 h	49	29.7 %	39	35.14 %	2.204	
	> 48 h	80	48.48 %	59	53.15 %	2.042	

COVID: Corona Virus Disease; OR: Odd Ratio; PCI: Percutaneous Coronary Intervention.

There was a consistent decrease of death during pandemic (OR 0.71, p 0.099), however it was statistically insignificant. We also found high likelihood of patient having complication such as cardiogenic shock (OR 1.49, p 0.045), TAVB (OR 2.66, p 0.015) and arrhythmia (OR 2.38, p 0.014) during COVID pandemic (Table 4).

**Table 4:** Multivariate analysis of outcomes before and during COVID pandemic.

	**OR**	**Lower (95** %**)**	**Upper (95** %**)**	**p-value**
Death	0.71	0.47	1.07	0.099
Cardiogenic shock	1.49	1.01	2.19	0.045
Heart failure	0.39	0.16	0.97	0.045
TAVB	2.66	1.2	5.85	0.015
Malignant arrhythmias	2.38	1.19	4.77	0.014

OR: Odd Ratio; TAVB: Total AV Block.

### 3.1 Subgroup analysis

Due to decreasing number of patients with several risk factor during COVID, we tried to perform analysis regarding correlation between traditional risk factors of CAD with mortality before and during pandemic (Supplementary Table 1); however, we found no correlation. On the other hand, death was more prevalent in FMC>48 h either before (OR 3.5, p < 0.001) or during COVID (OR 3.1, p 0.04) (Table 5).

**Table 5:** Distribution of death across FMC (all cases) before and during COVID.

FMC (hours)	Period	Total patient	Death (%)	OR	P value
0–2	Before COVID	376	12.2 %	Ref	
	During COVID	150	8.6 %		
2–6	Before COVID	217	11.5 %	0.934	0.796
	During COVID	96	11.4 %	1.364	0.471
6–12	Before COVID	102	6.8 %	0.529	0.125
	During COVID	53	7.5 %	0.86	0.80
12–48	Before COVID	84	11.9 %	0.969	0.933
	During COVID	41	9.7 %	1.139	0.828
>48	Before COVID	35	31.4 %	3.517	<0.001
	During COVID	22	22.7 %	3.1	0.044

COVID: Corona Virus Disease; FMC: First Medical Contact; OR: Odd Ratio.

## 4 Discussion

Although the effects of the pandemic have already lessened, the global health system is still focused on adapting to the COVID-19 outbreak as countries, including Indonesia, are still faced with thousands of daily cases [12]. The successful diagnosis and treatment of ACS is time dependent on many instances in the pre- and in-hospital settings, including FMC, diagnosis, and medical intervention [13]. Time management is crucial as ischemic time duration is a major determinant of infarct size, while prompt recognition and early management are critical in reducing morbidity and mortality [6], [13]. However, the sudden impact of the pandemic significantly increased the time to treatment in many different ways.

In our study, we found a reduction of 51.2 % in total patient two years after pandemic with similar presentation in age and gender. Similar reductions were shown by Mafham et al (40 %) and Spaccarotella et al (48 %) [9], [14]. This is considered a huge decrease compared to a study by Tern et al in Malaysia (23 %), Singapore (20 %) [15] and Clifford et al (20 %) [16]. These data consistent with meta-analysis by Sofi et al who documented a 20 % decrease in STEMI hospitalizations worldwide which also included a decrease > 50 % in their analysis thus highlighting the possibility of global underestimation of ACS cases during pandemic [17]. This could be related to massive “stay at home” campaigns from government and media outlets, added with increasing fear of COVID-19 infection which possibly leading to avoidance of hospitals even in the case of potential acute cardiovascular event [10].

In 1997, Weaver et al had already demonstrated that PCI was superior to thrombolysis for patients with acute myocardial infarction and is becoming a global recommendation nowadays [3], [6], [18]. This recommendation did not change even in the middle of COVID pandemic as ESC still prioritized primary PCI with an addition of 60 min delayed time due to COVID-19 related procedure [11]. However, the 60 min target is hardly achievable in many hospitals, including ours due to difference protocol in local guideline to maximize screening and prevent transmission of COVID-19. Additionally, many local recommendations implied administration of fibrinolysis initially while awaiting for COVID screening protocol [19], [20]. Jiang nan et al also stated that fibrinolytic therapy combined with deferred PCI can reduce ischemia time and has a similar in-hospital adverse outcome rate compared with patients who underwent primary PCI during the COVID-19 pandemic [21]. However, according to ESC STEMI guidelines from 2017, the most efficient agent in administration is Tenecteplase which can be given as a single bolus [3]. Unfortunately, the drug is not yet available in our country. In addition, during COVID, our health care resources were relocated to COVID-related post which made extra challenge in fibrinolysis. Hence, considering the procedure, observation time, complications, and human resources for fibrinolysis, it is considered inefficient to perform in patients with suspected COVID-19 in our hospital thus explaining the reduced rate of fibrinolysis (20.45 % vs 15.18 %, p value 0.054).

In our study, we found the number of patients undergoing PCI was increased during COVID (58.36 % vs 77.04 %, p value < 0,001); however, an increase in PCI rate should not be translated as achieving current recommendations as there was a significant decrease (43.41 % to 18.98 %, p < 0.001) in door to wire time before 12 h. Additionally, contrary to Tam et al who found a great difference in FMC during COVID-19 pandemic [22], we found no significant difference of FMC in all subgroup, highlighting in-hospital delays in our study during COVID as we must confirmed COVID status (clinical and/or laboratory screening) before proceeding due to lack of a dedicated COVID-19 catheterization laboratory. The whole process resulted in increased numbers of late presenter which also explained reduced rate of fibrinolysis and increased rate of PCI.

These findings of hospital delay probably translate into more complications such as cardiogenic shock (OR 1.49, p value 0.045), TAVB (OR 2.66, p value 0.015) and malignant arrhythmias (OR 2.38, p value 0.014) which we believe was due to prolonged ischemic time. These data are consistent with Erol et al which stated that major adverse cardiovascular event in ACS increase significantly after pandemic (4.8 % vs 8.9 %, p < 0.001) probably due to delayed treatment [7]. Uniquely, although statistically non-significant, we found less mortality during pandemic (OR 0.71, p value 0.099) which was unexpected. We believe that reduced mortality could be affected by a significant decrease in total patient, which we suspected that many “fatal” cases did not actually present at the hospital. This was consistent with a decrease in the percentage of Killip class II-IV patients in our study.

Although there were many studies highlighting the worsening effect of CAD’s traditional risk factor to outcomes [23], [24], [25], [26], we did not find significant correlation of traditional risk factor with death cases during our observation (Supplementary Table 1), strengthening our findings that in hospital delay was in fact the major problem in ACS even before and during COVID pandemic (Table 5).

Many similar studies have been published previously but we believe this is the first one in Indonesia reporting mortality in patients with ACS after COVID pandemic with the longest observation time. We believe the result of our study could add more information to global effect of COVID-19 pandemic and highlight the importance of aggressive patient education to lessen the impact of COVID-19 pandemic. Additionally, this “sudden” pandemic is a reminder that our health care system is fragile and we may be unprepared for similar events in the future. Thus, a back-up protocols are necessary.

The results of our study are limited by several factors. As the study is retrospective, we cannot specifically determine the cause of death, and other causes must be considered in this context. Second, although the province where the hospital is located has the highest positivity rate for COVID-19, our study was based at a single centre, and the results must be externally validated [12]. Third, although door to wire time was included in our study, we understand that deeper classification of PCI (primary, rescue, or routine PCI) could be done to better understand outcomes or complications, however during COVID, due to COVID related procedure and limited resources in our hospital, it was difficult to fit into definition stated by the guidelines. Fourth, in patient with fibrinolytics, we did not had door to needle time in our study as it massively affected by COVID related procedure. Finally, our study was only able to report in hospital mortality; as there was a decrease in total admissions during COVID, it is possible that the overall mortality is higher**.**

## 5 Conclusion

Residual effects of COVID-19 are still affecting our health care system. The huge decreases in total patients also mark that the healthcare system have not fully recovered despite transition from a pandemic to an endemic. The decrease in mortality is unexpected despite the findings of in-hospital delays thus a strategy should be prioritized, and revisions to the screening protocol are necessary to shorten the time to treatment of ACS. Although the COVID-19 situation already lessened, similar situations are possible in the future and back-up protocol should be established as this pandemic shown how unprepared the health care system in its current state. Further multi-centre studies to analyze the impact of COVID-19 should be performed to observe the true societal effect of the pandemic and increase the validity of the findings.

**Ethics Approval and Informed Consent**

This study was approved by local ethics committee (Hasan Sadikin General Hospital) and conducted in accordance with the fundamental principles of the Declaration of Helsinki.

### CRediT authorship contribution statement

**Ferdy Sanjaya:** Conceptualization, Writing – original draft, Data curation. **Miftah Pramudyo:** Conceptualization, Data curation, Writing – original draft, Writing – review & editing, Supervision. **Chaerul Achmad:** Conceptualization, Data curation, Writing – original draft, Writing – review & editing, Supervision.

## Declaration of Competing Interest

The authors declare that they have no known competing financial interests or personal relationships that could have appeared to influence the work reported in this paper.

## Data Availability

The datasets used and/or analysed during the current study are available from the corresponding author on reasonable request.
