# Supplementary material for: Statistical findings and outcomes of acute coronary syndrome patients during COVID-19 pandemic: A cross sectional study
Source: Int J Cardiol Heart Vasc. 2023 Apr 26;46:101213. doi: 10.1016/j.ijcha.2023.101213 (PMC10130330; doi:10.1016/j.ijcha.2023.101213)
Supplement: Supplementary data 1 [file mmc1.docx]

Supplementary Table 1. Correlation of traditional CAD risk factor to mortality between period

| Risk Factor | Period | Death | | | | P-value | OR |
| --- | --- | --- | --- | --- | --- | --- | --- |
|  |  | Yes | | No | |  |  |
| Dyslipidemia | Before COVID | 18 | 10.65% | 151 | 89.35% | 0.43 | 0.805 |
|  | During COVID | 7 | 13.21% | 46 | 86.79% | 0.320 | 1.557 |
| DM | Before COVID | 29 | 16.29% | 149 | 83.71% | 0.089 | 1.495 |
|  | During COVID | 11 | 13.92% | 68 | 86.08% | 0.132 | 1.773 |
| Hypertension | Before COVID | 73 | 13.75% | 458 | 86.25% | 0.153 | 1.383 |
|  | During COVID | 22 | 9.44% | 211 | 90.56% | 0.970 | 0.987 |
| Smoker | Before COVID | 53 | 10.11% | 471 | 89.89% | 0.006 | 0.56 |
|  | During COVID | 17 | 9.24% | 167 | 90.76% | 0.874 | 0.947 |
| History of Premature CAD | Before COVID | 9 | 10.98% | 73 | 89.02% | 0.654 | 0.847 |
|  | During COVID | 3 | 7.14% | 39 | 92.86% | 0.587 | 0.713 |

CAD: Coronary Artery Disease; COVID: Corona Virus Disease; OR: Odd Ratio
